# Isonicotinonitrile–4-methyl­benzoic acid (1/1)

**DOI:** 10.1107/S1600536811019799

**Published:** 2011-05-28

**Authors:** Xing-Wei Cai, Hong-Fei Lu

**Affiliations:** a School of Biological and Chemical Engineering, Jiangsu University of Science and Technology, Zhenjiang, Jiangsu 212003, People’s Republic of China

## Abstract

The title structure, C_6_H_4_N_2_·C_8_H_8_O_2_, is built up from an assembly of isonicotinonitrile and 4-methyl­benzoic acid mol­ecules and may be regarded as a co-crystal. The two planar mol­ecules [r.m.s. deviations of 0.002 (6) and 0.0028 (11) Å, respectively] are linked by O—H⋯N and C—H⋯O hydrogen bonds. They are nearly coplanar and only twisted from each other by a dihedral angle of 2.48 (6)°. In the crystal, the components are inter­connected by slipped π–π stacking [centroid–centroid distance = 3.6797 (11), slippage = 1.304 Å] and inter­molecular C—H⋯N inter­actions.

## Related literature

For the structures of related derivatives, see: Fu *et al.* (2009 ▶); Aminabhavi *et al.* (1986 ▶); Dai & Fu (2008*a* ▶,*b* ▶). For the graph-set theory, see: Etter *et al.* (1990 ▶); Bernstein *et al.* (1995 ▶).
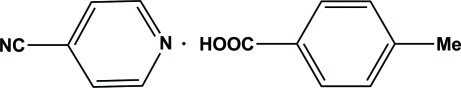


## Experimental

### 

#### Crystal data

C_6_H_4_N_2_·C_8_H_8_O_2_
*M* *_r_* = 240.26
Monoclinic, 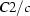

*a* = 7.5368 (15) Å
*b* = 13.049 (3) Å
*c* = 24.749 (5) Å
β = 94.20 (3)°
*V* = 2427.5 (8) Å^3^
*Z* = 8
Mo *K*α radiation
μ = 0.09 mm^−1^
*T* = 298 K
0.40 × 0.30 × 0.20 mm

#### Data collection

Rigaku Mercury2 diffractometer
Absorption correction: multi-scan (*CrystalClear*; Rigaku, 2005 ▶) *T*_min_ = 0.89, *T*_max_ = 1.00
11659 measured reflections
2752 independent reflections
2416 reflections with *I* > 2σ(*I*)
*R*_int_ = 0.033

#### Refinement

*R*[*F* ^2^ > 2σ(*F* ^2^)] = 0.041
*wR*(*F* ^2^) = 0.124
*S* = 1.09
2752 reflections
165 parameters
H-atom parameters constrained
Δρ_max_ = 0.28 e Å^−3^
Δρ_min_ = −0.18 e Å^−3^

### 

Data collection: *CrystalClear* (Rigaku, 2005 ▶); cell refinement: *CrystalClear*; data reduction: *CrystalClear*; program(s) used to solve structure: *SHELXS97* (Sheldrick, 2008 ▶); program(s) used to refine structure: *SHELXL97* (Sheldrick, 2008 ▶); molecular graphics: *ORTEPIII* (Burnett & Johnson, 1996 ▶) and *ORTEP-3 for Windows* (Farrugia, 1997 ▶); software used to prepare material for publication: *SHELXL97*.

## Supplementary Material

Crystal structure: contains datablocks I, global. DOI: 10.1107/S1600536811019799/dn2689sup1.cif

Structure factors: contains datablocks I. DOI: 10.1107/S1600536811019799/dn2689Isup2.hkl

Supplementary material file. DOI: 10.1107/S1600536811019799/dn2689Isup3.cml

Additional supplementary materials:  crystallographic information; 3D view; checkCIF report

## Figures and Tables

**Table 1:** Hydrogen-bond geometry (Å, °)

*D*—H⋯*A*	*D*—H	H⋯*A*	*D*⋯*A*	*D*—H⋯*A*
O1—H1⋯N1	0.82	1.87	2.6850 (15)	175
C1—H1*A*⋯O2	0.93	2.51	3.1858 (18)	130
C4—H4*A*⋯N2^i^	0.93	2.60	3.3700 (18)	141

Symmetry code: (i) 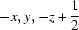.

**Fig. 1.**
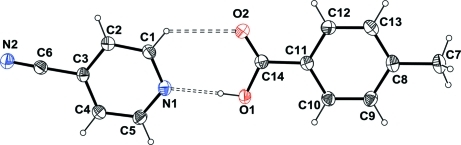
A view of the asymmetric unit with the atomic numbering scheme. The displacement ellipsoids were drawn at the 30% probability level. H atoms are represented as small spheres of arbitrary radii and the H bonds are shown as dashed lines.

## supplementary crystallographic information

### Comment

The amino derivatives have found wide range of applications in material science, such as magnetic, fluorescent and dielectric behaviors. And there has been an increased interest in the preparation of amino co-crystal compounds (Aminabhavi *et al.*, 1986; Dai & Fu 2008*a*; Dai & Fu 2008*b*; Fu, *et al.* 2009). As an extension on the structural characterization, we report here the crystal structure of the title compound isonicotinonitrile 4-methylbenzoic acid.

The asymmetric unit contains an organic isonicotinonitrile molecule and a 4-methylbenzoic acid organic molecule which are linked by a strong O—H···N and a weak C-H···O hydrogen bonds forming a C22(7) ring ( Etter *et al.*, 1990; Bernstein *et al.*, 1995)(Fig. 1). The benzene and pyridine rings are nearly coplanar and only twisted from each other by a dihedral angle of 2.48 (6)°. The geometric parameters of both the organic molecules are within the normal range.

There are intramolecular C-H···N hydrogen bonds and slippest π-π stacking which stabilize the packing (Tab.1 & 2).

### Experimental

isonicotinonitrile and 4-methylbenzoic acid were obtained commercially from Alfa Aesar. The two organoc compounds were solved in the solution (ethanol/water). Colourless block-shaped crystals suitable for X-ray analysis were obtained by slow evaporation of an ethanol/water (2:1 *v*/*v*) solution.

### Refinement

All the H atoms attached to C atoms were located into the idealized positions and treated as riding with C–H = 0.93 Å (aromatic) and 0.96 Å (methyl) with *U_iso_*(H)=1.2*U_eq_*(aromatic) and *U_iso_*(H)=1.5*U_eq_*(methyl). The positional parameters of the H atom (O1) was refined freely. In the last cycles of the refinement, it was treated as riding with the H1—O1 = 0.82 (2)Å) and *U_iso_*(H)=1.5*U_eq_*(O).

### Crystal data

**Table:**

C_6_H_4_N_2_·C_8_H_8_O_2_	*F*(000) = 1008
*M**_r_* = 240.26	*D*_x_ = 1.315 Mg m^−^^3^
Monoclinic, *C*2/*c*	Mo *K*α radiation, λ = 0.71073 Å
Hall symbol: -C 2yc	Cell parameters from 3221 reflections
*a* = 7.5368 (15) Å	θ = 3.1–27.5°
*b* = 13.049 (3) Å	µ = 0.09 mm^−^^1^
*c* = 24.749 (5) Å	*T* = 298 K
β = 94.20 (3)°	Block, colourless
*V* = 2427.5 (8) Å^3^	0.40 × 0.30 × 0.20 mm
*Z* = 8	

### Data collection

**Table:**

Rigaku Mercury2 diffractometer	2752 independent reflections
Radiation source: fine-focus sealed tube	2416 reflections with *I* > 2σ(*I*)
graphite	*R*_int_ = 0.033
Detector resolution: 13.6612 pixels mm^-1^	θ_max_ = 27.5°, θ_min_ = 1.7°
profile data from φ scans	*h* = −9→9
Absorption correction: multi-scan (*CrystalClear*; Rigaku, 2005)	*k* = −16→16
*T*_min_ = 0.89, *T*_max_ = 1.00	*l* = −32→32
11659 measured reflections	

### Refinement

**Table:**

Refinement on *F*^2^	Primary atom site location: structure-invariant direct methods
Least-squares matrix: full	Secondary atom site location: difference Fourier map
*R*[*F*^2^ > 2σ(*F*^2^)] = 0.041	Hydrogen site location: inferred from neighbouring sites
*wR*(*F*^2^) = 0.124	H-atom parameters constrained
*S* = 1.09	*w* = 1/[σ^2^(*F*_o_^2^) + (0.0713*P*)^2^ + 0.8361*P*] where *P* = (*F*_o_^2^ + 2*F*_c_^2^)/3
2752 reflections	(Δ/σ)_max_ = 0.001
165 parameters	Δρ_max_ = 0.28 e Å^−^^3^
0 restraints	Δρ_min_ = −0.18 e Å^−^^3^

### Special details

**Table:**

Geometry. All esds (except the esd in the dihedral angle between two l.s. planes) are estimated using the full covariance matrix. The cell esds are taken into account individually in the estimation of esds in distances, angles and torsion angles; correlations between esds in cell parameters are only used when they are defined by crystal symmetry. An approximate (isotropic) treatment of cell esds is used for estimating esds involving l.s. planes.
Refinement. Refinement of F^2^ against ALL reflections. The weighted R-factor wR and goodness of fit S are based on F^2^, conventional R-factors R are based on F, with F set to zero for negative F^2^. The threshold expression of F^2^ > 2sigma(F^2^) is used only for calculating R-factors(gt) etc. and is not relevant to the choice of reflections for refinement. R-factors based on F^2^ are statistically about twice as large as those based on F, and R- factors based on ALL data will be even larger.

### Fractional atomic coordinates and isotropic or equivalent isotropic displacement parameters (Å^2^)

**Table:**

	*x*	*y*	*z*	*U*_iso_*/*U*_eq_	
N1	0.43937 (13)	0.09948 (8)	0.42051 (4)	0.0221 (2)	
N2	−0.17678 (14)	0.10802 (9)	0.29801 (4)	0.0277 (3)	
C1	0.28921 (16)	0.13083 (9)	0.44087 (5)	0.0230 (3)	
H1A	0.2943	0.1523	0.4768	0.028*	
C2	0.12616 (16)	0.13288 (9)	0.41101 (5)	0.0234 (3)	
H2A	0.0238	0.1547	0.4264	0.028*	
C3	0.12108 (15)	0.10120 (8)	0.35729 (5)	0.0197 (3)	
C4	0.27629 (16)	0.06891 (10)	0.33537 (5)	0.0246 (3)	
H4A	0.2754	0.0473	0.2995	0.030*	
C5	0.43222 (16)	0.06995 (10)	0.36861 (5)	0.0256 (3)	
H5A	0.5369	0.0491	0.3542	0.031*	
C6	−0.04509 (16)	0.10367 (9)	0.32417 (5)	0.0224 (3)	
O1	0.73800 (11)	0.10321 (7)	0.48667 (3)	0.0269 (2)	
H1	0.6481	0.1058	0.4659	0.040*	
O2	0.55361 (11)	0.16298 (7)	0.54594 (4)	0.0289 (2)	
C7	1.30335 (18)	0.15462 (10)	0.69515 (5)	0.0299 (3)	
H7A	1.4122	0.1618	0.6776	0.045*	
H7B	1.3066	0.0923	0.7158	0.045*	
H7C	1.2898	0.2118	0.7189	0.045*	
C8	1.14836 (17)	0.15140 (9)	0.65291 (5)	0.0234 (3)	
C9	1.16972 (16)	0.11425 (9)	0.60095 (5)	0.0244 (3)	
H9A	1.2815	0.0926	0.5920	0.029*	
C10	1.02690 (16)	0.10904 (9)	0.56240 (5)	0.0227 (3)	
H10A	1.0432	0.0835	0.5280	0.027*	
C11	0.85908 (15)	0.14193 (8)	0.57500 (5)	0.0195 (3)	
C12	0.83696 (17)	0.17952 (9)	0.62657 (5)	0.0248 (3)	
H12A	0.7254	0.2016	0.6354	0.030*	
C13	0.98023 (18)	0.18425 (10)	0.66492 (5)	0.0272 (3)	
H13A	0.9636	0.2098	0.6993	0.033*	
C14	0.70140 (15)	0.13749 (9)	0.53480 (5)	0.0203 (3)	

### Atomic displacement parameters (Å^2^)

**Table:**

	*U*^11^	*U*^22^	*U*^33^	*U*^12^	*U*^13^	*U*^23^
N1	0.0212 (5)	0.0233 (5)	0.0215 (5)	−0.0027 (4)	0.0000 (4)	0.0016 (4)
N2	0.0229 (5)	0.0348 (6)	0.0251 (5)	0.0010 (4)	−0.0008 (4)	−0.0017 (4)
C1	0.0243 (6)	0.0241 (6)	0.0205 (5)	−0.0011 (4)	0.0001 (4)	−0.0021 (4)
C2	0.0216 (6)	0.0256 (6)	0.0229 (6)	0.0004 (4)	0.0010 (4)	−0.0021 (4)
C3	0.0201 (6)	0.0174 (5)	0.0212 (6)	−0.0025 (4)	−0.0009 (4)	0.0014 (4)
C4	0.0252 (6)	0.0292 (6)	0.0194 (5)	0.0010 (5)	0.0008 (4)	−0.0019 (4)
C5	0.0205 (6)	0.0330 (7)	0.0234 (6)	0.0017 (5)	0.0027 (4)	0.0007 (5)
C6	0.0233 (6)	0.0231 (6)	0.0210 (5)	−0.0005 (4)	0.0020 (5)	−0.0015 (4)
O1	0.0188 (4)	0.0422 (5)	0.0194 (4)	0.0009 (4)	−0.0011 (3)	−0.0046 (4)
O2	0.0200 (5)	0.0370 (5)	0.0295 (5)	0.0029 (4)	0.0000 (3)	−0.0074 (4)
C7	0.0301 (7)	0.0302 (7)	0.0279 (6)	−0.0046 (5)	−0.0085 (5)	0.0007 (5)
C8	0.0263 (6)	0.0208 (6)	0.0223 (6)	−0.0048 (4)	−0.0037 (5)	0.0030 (4)
C9	0.0196 (6)	0.0288 (6)	0.0249 (6)	−0.0008 (4)	0.0015 (4)	0.0019 (5)
C10	0.0217 (6)	0.0275 (6)	0.0189 (5)	−0.0023 (5)	0.0019 (4)	−0.0001 (4)
C11	0.0208 (6)	0.0177 (5)	0.0198 (5)	−0.0027 (4)	0.0009 (4)	0.0010 (4)
C12	0.0234 (6)	0.0259 (6)	0.0253 (6)	0.0006 (5)	0.0024 (5)	−0.0032 (5)
C13	0.0320 (7)	0.0289 (6)	0.0205 (5)	−0.0012 (5)	−0.0002 (5)	−0.0044 (5)
C14	0.0205 (6)	0.0186 (5)	0.0220 (6)	−0.0024 (4)	0.0018 (4)	0.0003 (4)

### Geometric parameters (Å, °)

**Table:**

N1—C1	1.3362 (16)	C7—C8	1.5100 (17)
N1—C5	1.3384 (16)	C7—H7A	0.9600
N2—C6	1.1461 (16)	C7—H7B	0.9600
C1—C2	1.3871 (17)	C7—H7C	0.9600
C1—H1A	0.9300	C8—C13	1.3903 (18)
C2—C3	1.3903 (16)	C8—C9	1.3946 (17)
C2—H2A	0.9300	C9—C10	1.3866 (17)
C3—C4	1.3903 (17)	C9—H9A	0.9300
C3—C6	1.4459 (17)	C10—C11	1.3925 (17)
C4—C5	1.3844 (17)	C10—H10A	0.9300
C4—H4A	0.9300	C11—C12	1.3886 (16)
C5—H5A	0.9300	C11—C14	1.4942 (16)
O1—C14	1.3202 (14)	C12—C13	1.3858 (18)
O1—H1	0.8200	C12—H12A	0.9300
O2—C14	1.2134 (15)	C13—H13A	0.9300
			
C1—N1—C5	118.32 (10)	H7B—C7—H7C	109.5
N1—C1—C2	123.14 (11)	C13—C8—C9	118.23 (11)
N1—C1—H1A	118.4	C13—C8—C7	120.95 (11)
C2—C1—H1A	118.4	C9—C8—C7	120.82 (11)
C1—C2—C3	117.72 (11)	C10—C9—C8	121.01 (11)
C1—C2—H2A	121.1	C10—C9—H9A	119.5
C3—C2—H2A	121.1	C8—C9—H9A	119.5
C4—C3—C2	119.88 (11)	C9—C10—C11	120.19 (11)
C4—C3—C6	120.28 (10)	C9—C10—H10A	119.9
C2—C3—C6	119.83 (11)	C11—C10—H10A	119.9
C5—C4—C3	117.85 (11)	C12—C11—C10	119.13 (11)
C5—C4—H4A	121.1	C12—C11—C14	118.85 (11)
C3—C4—H4A	121.1	C10—C11—C14	122.01 (10)
N1—C5—C4	123.09 (11)	C13—C12—C11	120.35 (11)
N1—C5—H5A	118.5	C13—C12—H12A	119.8
C4—C5—H5A	118.5	C11—C12—H12A	119.8
N2—C6—C3	178.44 (13)	C12—C13—C8	121.09 (11)
C14—O1—H1	109.5	C12—C13—H13A	119.5
C8—C7—H7A	109.5	C8—C13—H13A	119.5
C8—C7—H7B	109.5	O2—C14—O1	123.63 (11)
H7A—C7—H7B	109.5	O2—C14—C11	122.45 (10)
C8—C7—H7C	109.5	O1—C14—C11	113.91 (10)
H7A—C7—H7C	109.5		

### Hydrogen-bond geometry (Å, °)

**Table:**

*D*—H···*A*	*D*—H	H···*A*	*D*···*A*	*D*—H···*A*
O1—H1···N1	0.82	1.87	2.6850 (15)	175
C1—H1A···O2	0.93	2.51	3.1858 (18)	130
C4—H4A···N2^i^	0.93	2.60	3.3700 (18)	141

Symmetry codes: (i) −*x*, *y*, −*z*+1/2.

### Table 2 Table 2 π-π stacking interactions (Å,°)

Cg1 is the centroid of the C8—C13 ring.Cg2 is the centroid of the N1—C5 ring

**Table:**

CgI	CgJ	CgI···CgJ^a^	CgI···P(J)^b^	CgJ···P(I)^c^	Slippage
Cg1	Cg2^ii^	3.6797 (11)	3.440	3.443	1.304

Symmetry codes: (ii)3/2-x,1/2-y,1-z Notes:a : Distance between centroidsb : Perpendicular distance of CgI on ring plan Jc : Perpendicular distance of CgJ on ring plan ISlippage = vertical displacement between ring centroids.

## References

[bb1] Aminabhavi, T. M., Biradar, N. S. & Patil, S. B. (1986). *Inorg. Chim. Acta*, **125**, 125–128.

[bb2] Bernstein, J., Davis, R. E., Shimoni, L. & Chang, N.-L. (1995). *Angew. Chem. Int. Ed. Engl.* **34**, 1555–1573.

[bb3] Burnett, M. N. & Johnson, C. K. (1996). *ORTEPIII* Report ORNL-6895. Oak Ridge National Laboratory, Tennessee, USA.

[bb4] Dai, W. & Fu, D.-W. (2008*a*). *Acta Cryst.* E**64**, m1016. 10.1107/S1600536808019168 PMC2961939 21203009

[bb5] Dai, W. & Fu, D.-W. (2008*b*). *Acta Cryst.* E**64**, m1017. 10.1107/S1600536808006454 PMC2961940 21203010

[bb6] Etter, M. C., MacDonald, J. C. & Bernstein, J. (1990). *Acta Cryst.* B**46**, 256–262. 10.1107/s0108768189012929 2344397

[bb7] Farrugia, L. J. (1997). *J. Appl. Cryst.* **30**, 565.

[bb8] Fu, D.-W., Ge, J.-Z., Dai, J., Ye, H.-Y. & Qu, Z.-R. (2009). *Inorg. Chem. Commun.* **12**, 994-997.

[bb9] Rigaku (2005). *CrystalClear* Rigaku Corporation, Tokyo, Japan.

[bb10] Sheldrick, G. M. (2008). *Acta Cryst.* A**64**, 112–122. 10.1107/S0108767307043930 18156677

